# Deaminated purine bypass by DNA polymerase η

**DOI:** 10.1042/BCJ20200989

**Published:** 2021-03-29

**Authors:** Antonina Andreeva

**Affiliations:** MRC Laboratory of Molecular Biology, Francis Crick Avenue, Cambridge CB2 0QH, U.K.

## Abstract

A recent work by Jung and colleagues (*Biochem J.* **477**, 4797–4810) provides an explanation of how DNA polymerase η replicates through deaminated purine bases such as xanthine and hypoxanthine. This commentary discusses the crystal structures of the polymerase η complexes that implicate the role of tautomerism in the bypass of these DNA lesions.

Genomic DNA of living cells is frequently exposed to a variety of endogenous or exogenous factors that can lead to its damage. The damaged DNA is an obstacle for the replication machinery and it usually results in a replication block due to the inability of the classical high-fidelity DNA polymerases to incorporate nucleotide opposite to the DNA lesion. This block can cause replication fork collapse leading to DNA breaks, chromosomal rearrangements and ultimately to a cell death. To maintain genomic stability, cells have evolved different mechanisms to either repair, or tolerate and bypass the site of DNA damage. The latter mechanism involves the temporary replacement of the replicative polymerase by one or more specialized translesion synthesis polymerases that are able to replicate through the damaged bases, typically in an error-prone manner [1]. In eukaryotes, the DNA polymerases that mediate the translesion synthesis are members of the so-called Y-family that include polymerase eta, kappa, iota and REV1. They exhibit different specificity and fidelity in the lesion bypass owing to the differences in their active site architectures. The Y-family polymerases share similar organization consisting of an N-terminal catalytic core followed by a flexible intrinsically disordered region and a set of regulatory protein–protein interaction modules toward the C-termini. Architecturally the catalytic core is reminiscent to the classical polymerases in which the palm domain harbours the residues crucial for catalysis, whereas the thumb and finger domains grasp the DNA and make contacts with the primer and the template, respectively. The structural similarity between the classical replicative and the Y-family lesion-bypass polymerases is, however, limited to the palm domains that superpose quite well with their functional residues at equivalent conserved positions. A distinct feature of the Y-family is the presence of a little finger domain, also known as polymerase associated domain (PAD), that is unique to this family of polymerases. This domain makes additional contacts with the DNA and is implicated in the ternary complex stability.

The most intriguing and probably best-studied member of the Y-family is the human polymerase eta (hPolη). This enzyme has the unique ability to efficiently replicate through UV-induced cis-syn thymine-thymine (T-T) dimers in an error-free manner by inserting AA opposite TT. The deficiency in this polymerase is associated with a human disorder xeroderma pigmentosum, which increases the risk of developing skin and other internal cancers. hPolη also facilitates the translesion synthesis of cancer drug adducts, such as cisplatin and phenanthriplatin, that can lead to impaired response to cancer treatment and development of cancer chemoresistance. In addition to the above, the enzyme has been implicated in the bypass of various mutagenic DNA lesions, including O6-methyl-2′-deoxyguanosine (O6-MeG), 7,8-dihydro-8-oxo-2′-deoxyguanosine (8-oxoG), 8,5′-cyclo-2′-deoxyadenosine (cdA), abasic sites (AP) etc. Over the past decade, the crystal structures of the hPolη replicative bypass of these lesions have been determined, revealing the intimate mechanistic details of the polymerase function [2–4]. Two new crystal structures reported in the recent issue of *Biochemical Journal* by Jung et al. [5] expand the broader work on human Polη substrates and provide the first structural insights into the mutagenic bypass of deaminated purine lesions. These structures show the ternary complex of the polymerase with a template DNA containing either xanthine (XT) or hypoxanthine (HX) lesion in the active site and a non-hydrolyzable dCMPNPP (dCTP*) as an incoming nucleotide. In both structures, the templating XT/HX is positioned between the finger and little finger domains, whereas the incoming dCTP* is nested between the palm and finger domains. The Polη-XT:dCTP* and Polη-HX:dCTP* complexes reveal significant similarity to the Polη-dG:dCTP* complex with undamaged DNA and to other hPolη structures inserting dCTP* opposite a damaged base, suggesting that the polymerase and its substrates undergo little conformational changes (Figure 1). Some key elements such as the side-chain conformation of the active site residues, the binding of the metals, the position of the primer 3′-OH and the 5′ α-phosphate of the incoming dCTP* are essentially the same in these structures. An exception makes the side chain of Arg61 that displays some conformational flexibility. This residue is located at the tip of the finger domain and its dynamics and importance for the polymerase activity has been extensively discussed previously [2–4]. In both, Polη-XT:dCTP* and Polη-HX:dCTP* complexes, Arg61 is involved in a cation-π stacking interaction with the incoming dCTP* and thereby supporting its optimal position for base pairing. The guanidino group of this residue swivel over the nucleotide and either points toward the phosphate groups of dCTP* (in Polη-HT:dCTP*) or toward the template DNA thereby making a close contact to O4 of dT(N + 1) base (in Polη-XT:dCTP*). Another significant conformational difference resides in the template DNA, in particular the region upstream of the insertion site. While in Polη-XT:dCTP* complex the dT(N + 1) and dA(N + 2) bases are displaced toward the active site interior, in Polη-HX:dCTP*, the dT(N+1) base flips toward the exterior. It looks quite plausible that the interplay between the flexibility of the Arg61 side-chain and the conformational dynamics in the DNA substrate may have an impact on the polymerase fidelity and catalytic efficiency, and can contribute to the kinetic differences reported in this study [5].

**Figure 1.**
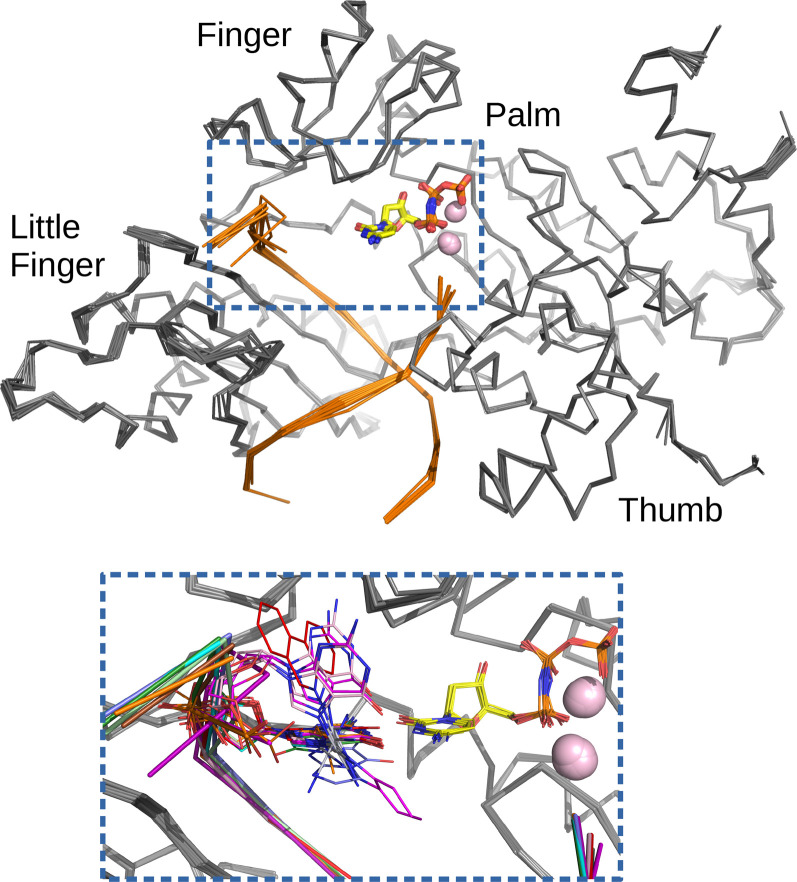
Structural comparison of hPolη catalytic core complexes with DNA containing different lesions and dCTP* as an incoming nucleotide. Top: Backbone superposition of the polymerase complexes. The enzyme and DNA are shown in ribbon, dCTP* in sticks and Mg atoms as spheres and coloured in grey, orange, yellow and pink, respectively. The superposition includes the following hPolη structures inserting dCTP* opposite: oxaliplatin-GpG (6MXO), phenanthriplatin-dG (4Q8E), hypoxanthine (6MQ8), xanthine (6WK6), 1,N2-ɛdG (5F9N), MeFapy-dG (4RU9), Fm7dG (6UI2), cis-Pt-1,2-d(GpG) (4DL4), 8-oxoG (4O3P), undamaged DNA (4O3N). Bottom: Detailed view of the hPolη active site (lesions shown in a different colour).

Xanthine and hypoxanthine are major lesions formed by the deamination of guanine and adenosine, respectively, and are both highly promutagenic. The steady-state kinetic data presented by Jung and colleagues suggests that Polη can correctly incorporate dCTP opposite xanthine, albeit marginally more efficient than dTTP. In contrast, the presence of templating hypoxanthine significantly facilitates the insertion of dCTP instead of dTTP and thus promoting post-replicative A to G mutation. How can hPolη correctly insert cytosine opposite xanthine? In the crystal structure, the O2 of xanthine is in a proximity (2.7 Å) to O2 of dCTP* and this can create a strong electrostatic clash between the carbonyl groups (Figure 2A). The bases of XT:dCTP* pair, however, lie in a common plane, with the distance between C1′ sugar atoms ∼10.4 Å and the C1′–C1′ vector forming similar angles with the xanthine C1′–N9 and cytosine C1′–N1 glycosidic bonds, suggesting that xanthine and dCTP* base pair with Watson–Crick geometry. At neutral pH, xanthine exists as a mixture of two tautomeric forms at the 2-position: a 2,6-keto and a 6-keto-2-enol tautomer. Only the enol tautomer contains a hydroxyl group that can form a hydrogen bond with the carbonyl from the dCTP* and satisfy the hydrogen bonding requirements, suggesting that O2-enol tautomer of xanthine is involved in the base pairing. Thus the tautomeric form of the XT:dCTP* pair stereochemically mimics the canonical G:dCTP pair in the insertion site and facilitates the correct nucleotide insertion (Figure 2A,B). It is likely that the polymerase active site local environment plays a role in perturbing the tautomeric equilibria and stabilizing the O2-enol form of xanthine. In part, this can be attributed to Gln38 that can establish a hydrogen bond with N3 of xanthine. The solvent-accessibility and water network of the polymerase active site can also contribute to its stabilization. The stabilization of enol tautomer form of a nucleobase has been observed in other base-pairs and RNA-ligand interactions. For example, xanthine binds to guanine-responsive riboswitch as a O2-enol tautomer and similarly base pairs with cytosine. Moreover, the crystal structures of riboswitch bound to xanthine or guanine revealed no apparent differences between xanthine and guanine binding [6].

**Figure 2.**
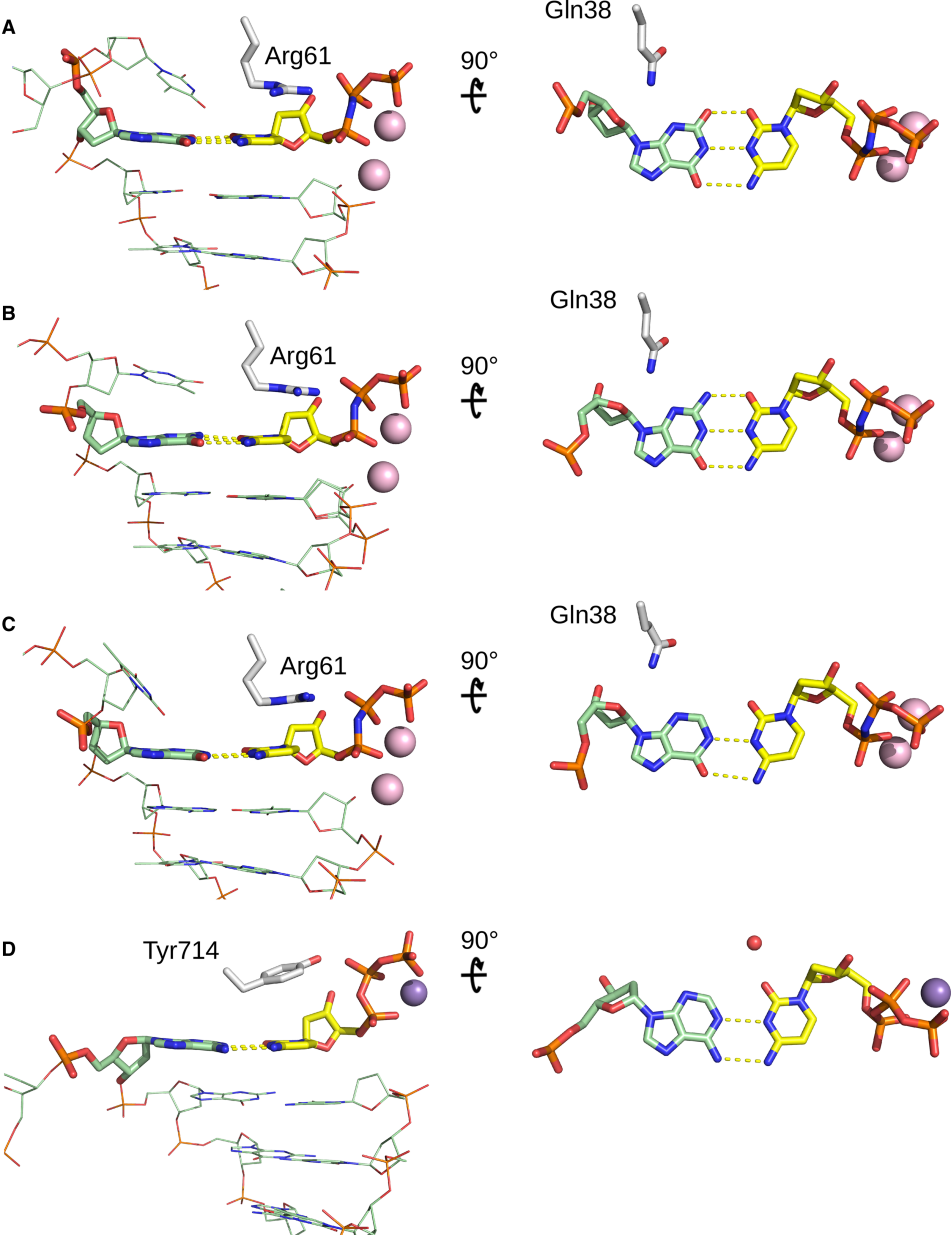
Active site base pairing in the DNA polymerase η complexes. The left panel shows a view from the major groove and the right panel shows a view from the top after ∼90° rotation. (**A**) XT:dCTP* (6WK6); (**B**) G:dCTP* (4O3N); (**C**) HT:dCTP* (6M8Q); DNA is shown in green, dCTP* in yellow and Mg atoms in pink. (**D**) Active site base pairing in the high-fidelity DNA polymerase I complex containing A:dCTP mismatch (3PX0). DNA and dCTP shown in green and yellow, Mn atom in purple and the water molecule implicated in the stabilization of the rare imino cytosine tautomer is shown in red.

While hPolη is capable of bypassing xanthine in an error-free manner, the bypass of hypoxanthine leads to a misincorporation of cytosine. Hypoxanthine does not have 2-amino group but the 6-keto and N1 groups can potentially engage in a Watson–Crick type of base pairing. Indeed, in the Jung and co-workers’ structure, hypoxanthine and dCTP* form a base pair with Watson–Crick geometry stabilized by two hydrogen bonds, suggesting that O6-keto tautomer of hypoxanthine is involved in the pairing. The HT:dCTP* pair, similar to the XT:dCTP*, sterically resembles the canonical G:dCTP base pairing in the hPolη active site (Figure 2B,C). Interestingly, hypoxanthine also binds to guanine-responsive riboswitch and base pairs with cytosine in the riboswitch binding pocket with a similar hydrogen-bonding pattern [7].

Rare tautomeric nucleobases and nucleobase analogs play an important part in DNA damage and repair, mutagenesis, nucleic acid recognition, RNA-ligand recognition and catalysis. For example, mismatched base pairs that could involve minor tautomeric forms were observed in the near-cognate codon–anticodon helices in the decoding center of the 70S ribosome [8]. Furthermore, several recent structures of high-fidelity DNA polymerases provide strong support to the role of tautomerism in the replication infidelity with the observation of incorrect base pairs forming Watson−Crick-like geometries in the enzyme active site and thus mimicking the stereochemical features of their canonical substrates. Such base pairs were observed in the crystal structure of a human DNA polymerase λ poised to misinsert dGTP opposite a template T [9]. Similarly, the structures of DNA polymerase I containing in the active site mismatched O6MeG:ddCTP [10] and A:dCTP [11] base pairs, were consistent with the presence and stabilization of a rare cytosine tautomer (Figure 2D). The work by Jung and colleagues demonstrates another example of how tautomeric forms of the deaminated purines, xanthine and hypoxanthine, base pair in the DNA Polymerase η active site and can contribute to the error-free and error-prone bypass of these DNA lesions.

Despite the growing structural and biochemical evidence of the important roles of nucleobase tautomers, their occurrence, stability and mechanisms are still not fully understood. Tautomerism involves the movement of protons that are difficult to evidence experimentally with the common techniques such as X-ray crystallography and cryo-EM. At average resolution, it is not possible to unambiguously resolve the positions of protons and to distinguish between different tautomeric forms. These challenges highlight the need for the development of new biophysical approaches that can be applied to examine and better understand the roles of tautomerism in biological systems and processes.

## Abbreviations

1,N2-ɛdG: 1,N2-etheno(ɛ)deoxyguanosine
8-oxoG: 7,8-dihydro-8-oxo-2′-deoxyguanosine
AP: abasic sites
Fm7dG: N7-methyl-2-deoxyguanosine
HX: hypoxanthine
MeFapy-dG: N(6)-(2-Deoxy-D-erythro-pentofuranosyl)-2,6-diamino-3,4-dihydro-4-oxo-5-N-methylformamidopyrimidine
O6MeG: O6-methyl-2′-deoxyguanosine
PAD: polymerase associated domain
XT: xanthine
